# Establishment of Simple and Routine Methods in Early Diagnosis of Gentamicin-Induced Kidney Injury Based on a Rat Model

**DOI:** 10.1155/2016/7160903

**Published:** 2016-08-18

**Authors:** Cuiyan Liu, Youxi Kang, Huiqin Zhang, Long Zhu, Hai Yu, Chunyang Han

**Affiliations:** College of Animal Science and Technology, Anhui Agricultural University, Hefei, Anhui 230036, China

## Abstract

The changes in biomarkers of gentamycin- (GM-) induced kidney injury have been studied by using simple and routine methods and also assessed the efficacy and utility of these routine biomarkers in early diagnosis. Eighty Sprague-Dawley (SD) rats were randomly divided into 4 groups: three experimental groups treated with different GM dosages (4, 20, and 100 mg·kg^−1^) and a control group. The experimental groups were given intramuscular GM injections once daily for 14 days, and the control group was given intramuscular sterile water. Blood and urine samples were collected on treatment days 1, 3, 7, and 14 to test for total protein (TP), albumin (ALB), blood urea nitrogen (BUN), creatinine (CRE), uric acid (UA), pH, specific gravity (SG), proteins (PRO), and cells in urinary sediment. Histopathology and kidney coefficient were performed on excised kidney specimens. The result indicated that serum CRE, BUN, and TP, urine PRO, and urinary hyaline casts and low-transitional epithelium showed an immediate and highly sensitive response to kidney injury, and the combined diagnosis with the above methods could be used in early diagnosis. Particularly, the process of the test was simple and quick, no special equipment, so it is more suit for primary medical institution.

## 1. Introduction

Gentamicin (GM) is an aminoglycoside that is a highly effective antibiotic agent, especially in Gram-negative infections [1]. However, large doses or long-term use of GM can induce kidney injury [2–5]. As the kidney is a vascular organ, renal tissue damage can easily cause bleeding and urine leakage, thereby resulting in local or systemic infection and even shock [6]. Thus, regular monitoring for drug-induced kidney injury during GM treatment is important for the rational use of GM and early diagnosis.

Biomarkers [7] of kidney injury include, among others, blood urea nitrogen (BUN), creatinine (CRE), uric acid (UA), total protein (TP), and protein (PRO). Currently, in nonclinical drug-safety evaluation and clinical diagnosis, BUN and CRE are frequently used as renal-injury biomarkers; however, both CRE and BUN levels can be affected by several factors other than renal dysfunction, and hence, their sensitivities were limited only to a certain degree of renal injury [8]. In contrast, PRO, UA, and cells in urinary sediment can reveal obvious changes in GM-induced kidney injury in rats; thus, they can be used as important parameters for disease diagnosis [9–11]. In this study, we discuss the changes and differences in biomarkers brought about by dose and duration-dependent GM-induced kidney injury. We employed simple and convenient biochemistry methods to provide a reference for early diagnosis and real-time monitoring of kidney injury in clinic.

## 2. Materials and Methods

### 2.1. Animals

In total, eighty 5-week-old, adult, male Sprague-Dawley rats (weight, 180–200 g) were obtained from Aier Matt Technology Co., Jiangsu Province, China (animal license: SCXK (Su) 2014-0007). The animals were maintained in standard housing facilities (temperature: 24 ± 1°C, humidity: 45 ± 5%, and a 12-h light/dark cycle) and fed standard laboratory chow, with* ad libitum* access to water. All animals were given a week to acclimatize before the start of GM treatment. All procedures were in strict accordance with the Chinese legislation on the use and care of laboratory animals and the guidelines established by Institute for Experimental Animals of Anhui Agriculture University and were approved by Anhui Agriculture University Committee on Animal Care and Use.

### 2.2. Animal Groups and Experimental Design

After acclimatizing for 1 week, the 80 rats were randomly divided into 4 groups (*n* = 20 each): 3 experimental groups treated with different dosages of GM (4, 20, and 100 mg·kg^−1^) and a control (sterile water) group. Gentamicin sulfate was purchased from Lianshui Pharmaceutical Company, Jiangsu Province, China. There were no significant differences within and between groups with respect to weight and quantities of feed and drinking. Experimental groups were given intramuscular GM injections once daily for 14 days, and the control group was given intramuscular sterile-water injections. Body weight and feed and water intakes were recorded every day. Urine and blood samples were collected on days 1, 3, 7, and 14 of treatment. Serum samples used to detect total protein (TP), albumin (ALB), blood urea nitrogen (BUN), creatinine (CRE), uric acid (UA), and urine samples were used to detect the pH, specific gravity (SG), protein (PRO), and cells in urinary sediment. And five rats from each group were randomly chosen, and their kidneys were excised for kidney coefficient and histopathology examination.

### 2.3. Blood Biomarkers Detection (TP, ALB, CRE, BUN, and UA)

After fasting for 12 h, 3–5 mL blood was drawn from each rat's caudal vein. Samples were centrifuged at 2500 rpm for 5 min to separate the serum. Finally, serum levels of TP, ALB, CRE, BUN, and UA were detected by CelercareV1 Automatic Biochemistry Analyzer (Weinarui Technology Co., Tianjing, China). All detection kits were purchased from Jiancheng Bioengineering Institute production, Nanjing, Jiangsu Province, China.

### 2.4. Urine Routine Detection (pH, SG, and PRO)

Using the bladder oppression method, we collected 3–5 mL urine from each rat. In all, 2 mL urine was used for detection of the abovementioned parameters on HY-632 Automatic Urine Chemistry Analyzer (Huiyan Kechuang Biotechnology Co., Shenzhen, Guangdong Province, China).

### 2.5. Urinary Sediment Detection

For preparation of Sternheimer dyeing liquor, 2% Alcian blue 8GX (Meilun Biological Technology Co., Dalian, Liaoning Province, China) and 1.5% Pyronine B (Yuanye Biological Technology Co., Shanghai, China) were mixed in a 2 : 1 ratio by HYQ-3110 Vortex Mixers (Jingqi Limited Company, USA) (to be used within a month) and filtrated. For detection of urinary sediments, we centrifuged 1-2 mL urine with 1500 rpm for 5 min. After discarding the supernatant, 2 drops of the urinary sediment were mixed with 1 drop of the Sternheimer dyeing liquor. This was allowed to stand for 5–10 min, after which a drop of the mixed solution was mounted onto glass slides and examined under a microscope (CX21FS1 Biological Microscope; OLYMPUS Co., Japan).

### 2.6. Kidney Coefficient and Histopathological Examinations

The kidney tissues of the rats were weighed to calculate the kidney coefficient (kidney coefficient = kidney weight/body weight) and then fixed in a 10% buffered formalin solution. The tissues were subjected to standard alcohol–xylol processes and embedded in paraffin. The samples were cut into 5 *μ*m thick sections (LS-2055+ Paraffin Semiautomatic Machine; Longshou Electronic Instrument Co., Shenyang, Liaoning Province, China) and stained with hematoxylin and eosin (HE). Kidney sections were examined by light microscopy (CX21FS1 Biological Microscope; OLYMPUS Co., Japan) and assessed at 20x magnification by randomly selecting areas in the microscopic field at 10x, to determine changes in degeneration and necrosis.

### 2.7. Statistical Analysis

The data were analyzed with ANOVA and Dunnett's test using the SPSS package program (v 19.0; IBM, USA). Results of all groups are shown as mean values ± SD. The association between two groups was determined by Dunnett's multiple comparison test.* P* < 0.05 was considered statistically significant.

## 3. Results

### 3.1. Effect of GM on Mortality, Body Weight, and Feed and Water Intake

#### 3.1.1. Mortality

All animals in the 100 mg·kg^−1^ GM group died on the 20th day of the experiment (i.e., on day 13 of starting GM treatment). No deaths were observed in the other 3 groups.

#### 3.1.2. Body Weight

**Figure 1:**
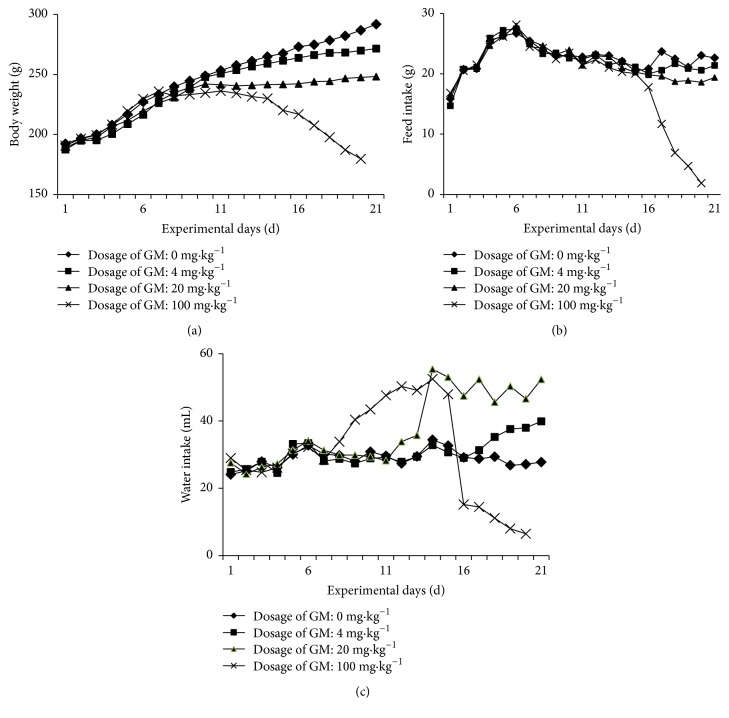
Body weight (a), feed intake (b), and water intake (c) of rats with different dosage and duration of experiment.

The body weight of rats increased in all groups before the 10th day (i.e., on day 3 of starting GM treatment). Subsequently, the growth rate of the animals' body weights in the experimental groups decreased with the increase of GM dosage and duration (Figure 1(a)).

#### 3.1.3. Feed Intake

Changes in feed intake were closely related to changes of body weight; feed intake of rats decreased with the increase of GM dosage and duration. On the 15th day (i.e., on day 8 of starting GM treatment), the feed intake of rats in the 100 mg·kg^−1^ GM group significantly decreased (*P* < 0.05) (Figure 1(b)).

#### 3.1.4. Water Intake

Water intake of rats in the 4 mg·kg^−1^ GM group increased on the 16th day (i.e., on day 9 of starting GM treatment); that in the 20 mg·kg^−1^ GM group increased on the 11th day (i.e., on day 5 of starting GM treatment); and that in the 100 mg·kg^−1^ GM group increased on the 8th day (i.e., on day 1 of starting GM treatment) and sharply declined on the 16th day (i.e., on day 9 of starting GM treatment). All results are in comparison to the control group (Figure 1(c)).

### 3.2. Changes in Serum Biomarkers

On day 1 of GM treatment, serum levels of TP and ALB in the experimental groups were significantly lower than those of the control group (*P* < 0.05), and CRE and BUN were significantly higher (*P* < 0.05). On day 3, TP, ALB, CRE, BUN, and UA showed significant differences between the experimental and control groups (*P* < 0.05), and CRE was significantly higher than the other 2 experimental groups (*P* < 0.05); on day 7, TP, CRE, and BUN showed significant differences among all groups (*P* < 0.05) (Table 1).

### 3.3. Changes in Urine Biomarkers

The pH of urine reduced (*P* < 0.05) in the 100 mg·kg^−1^ GM group on day 7 of treatment, while SG remained largely unchanged with respect to dosage and duration of treatment (*P* > 0.05) (Table 2). Both the numbers and degree of PRO positivity increased proportionally with the dosage and duration of GM treatment; this was particularly notable in the 100 mg·kg^−1^ GM group (Table 3).

**Figure 2:**
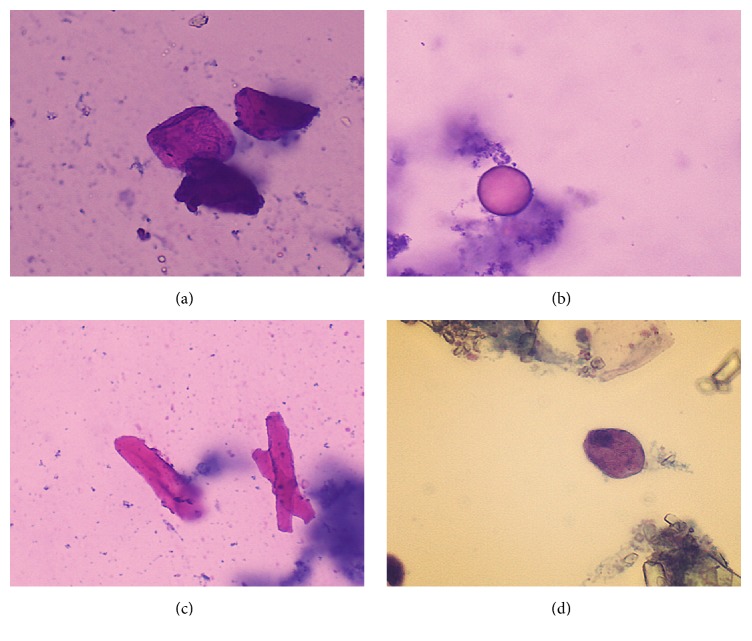
Pavement epithelium cells (a), red blood cells (b), hyaline cast (c), and low-transitional epithelium (d) in urinary sediment of rats with GM-induced kidney injury (hematoxylin and eosin, 200x).

While pavement epithelium cells could also be found in healthy urine samples, they increased in numbers upon kidney injury (Figure 2(a)). Red blood cells (Figure 2(b)), hyaline cast (Figure 2(c)), and low-transitional epithelium (Figure 2(d)) could be found on day 3 of GM treatment, and they increased proportionally with dosage and duration of treatment.

### 3.4. Changes in Kidney Coefficient and Histopathology

The kidney coefficient showed no significant difference among groups (*P* > 0.05) (Table 4).

**Figure 3:**
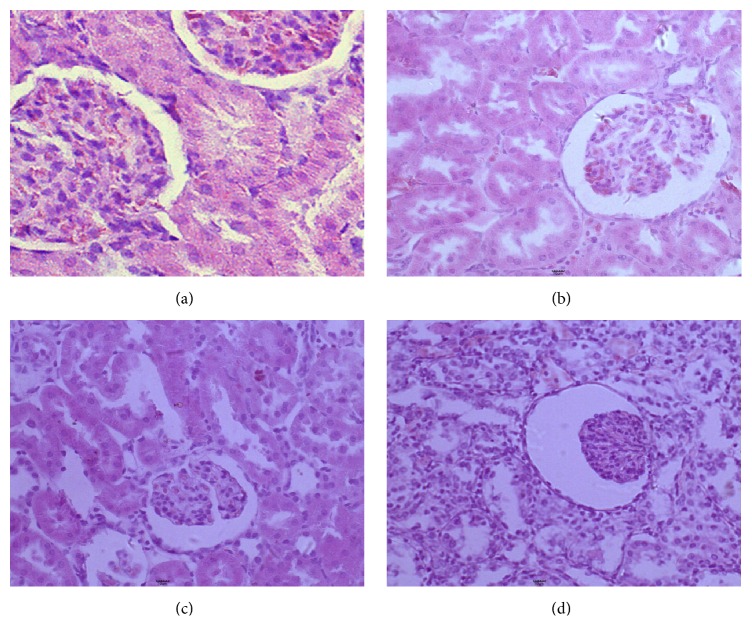
Pathological changes in kidney of rats in the 100 mg·kg^−1^ GM group on days 1 (a), 3 (b), 7 (c), and 14 (d) after start of treatment (hematoxylin and eosin, 200x).

Pathological changes of renal tissue were observed by HE staining. In control group, the structure of the kidney was complete, and the structures of glomerular and tubular structures were normal (Figure 3(a)). In 4 mg·kg^−1^ GM group, during the whole research period, there are not significant pathological changes of kidney with visible swelling of renal tubular epithelial cells. In 20 mg·kg^−1^ GM group, on day 7 of GM treatment, tubular lumen narrowing and a slight congestion could be found, and ond 14, a great quantity of epithelial cells swelled and ruptured. In 100 mg·kg^−1^ GM group, on day 3, visible swelling of renal tubular epithelial cells, granular degeneration, tubular lumen narrowing, and a slight congestion could be found in the 100 mg·kg^−1^ GM group (Figure 3(b)). On day 7, the epithelial cells of renal tubules were vacuolar degeneration, and a large number of epithelial cells swelled and ruptured (Figure 3(c)). On day 14, partial renal tubular structure is not complete with glomerulus shrinked, and it is obvious in renal interstitial fibrosis (Figure 3(d)).

## 4. Discussion

Kidney injury is a syndrome performancing as renal excretion, glomerular filtration function decreasing, and imbalance of water, electrolyte, and acid-base caused by a variety of pathogenic factors [12]. Conventionally accepted kidney-injury biomarkers include BUN, CRE, UA, PRO, and TP. BUN is an important biomarker of glomerular filtration, and BUN levels would increase when glomerular cells were damaged and the glomerular filtration rate (GFR) reduced [13]. CRE is an important reference parameter of glomerular filtration, and increased levels of CRE indicate kidney injury [14]. All UA was filtered by the glomerulus with tubular reabsorption of most UA. Levels of UA are found in excess when there is a dysfunction in the mechanism of UA excretion or there was too much UA buildup for timely and efficient excretion. Suppression of renal tubular secretion, increase of tubular resorption, and decrease of glomerular filtration all contribute to increasing levels of UA [15]. The level of TP mainly reflects the loss of protein caused by liver synthesis and renal diseases. Proteins in urine were primarily related to the filtration barrier of the renal glomerulus function; moreover, PRO in urine were considered to be good indicators of early kidney disease, and high concentration of PRO will induce more serious kidney disease [16]. As reported previously [17], small changes in kidney function led to accumulation of hyaline casts and low-transitional epithelium in urine, while accumulation of low-transitional epithelium would indicate marked pathological changes. There are reports [5, 18–22] that kidney injury induced by gentamicin induced proteinuria, cylindruria, and metabolic disorders of BUN, CRE, TP, and so forth; histological findings showed that the epithelial cells of renal tubular epithelial cells were swollen and detached; renal function damage occurs with severe kidney injury.

Our study investigated the biochemical changes in kidney-injury biomarkers such as BUN, CRE, UA, PRO, and cells in urinary sediment in early diagnosis GM-induced kidney injury in rats. TP, ALB, CRE, and BUN in the experimental groups showed significant differences (*P* < 0.05) on day 1 of GM treatment as compared to the control group, and after day 3, these biomarkers showed significant differences among the different groups. Changes of the above indices are consistent with the performance of renal injury [12–16]. PRO in urine samples of the experimental group showed positivity on day 1 of treatment, and the number and degree of PRO positivity positively correlated with the duration and dosages of GM treatment. In urinary sediment, from day 3 of treatment, pavement epithelium cells, red blood cells, hyaline casts, and low-transitional epithelium could be found, which proportionally increased in number with GM dosage and duration. Above results were similar to Prescott and Brodie's result [17]. SG showed no significant differences (*P* > 0.05) throughout the experiment. SG could be analyzed with urinary volume [21]. The pH of urine reduced (*P* < 0.05) in the 100 mg·kg^−1^ GM group on day 7. The increasing acidity of urine was consistent with the degree of kidney injury [22]. Rats in the 100 mg·kg^−1^ GM group died on day 14, and in histopathological examinations, severe kidney lesions were found that most renal cells showed swollen and detached, renal interstitial fibrosis, and complete loss of cellular integrity. The result is similar to those that have been well documented [23]. Without significant pathological changes of other organs, kidney injury may be leading cause of death. With the obvious pathological changes and the increase of dosage, the degree of kidney injury increased, which was the reason why feed intake reduced; body weight of rats grew slowly, even showing loss of weight with roughened hair coat and mental fatigue, which was closely linked to reduction of feed intake reduced by kidney injury; water intake increased obviously, and degree and time of increase were different with dosages, and this might be led to the changes of release of antidiuretic hormone (ADH), activity of thirst center in the cerebral cortex, and thirst sensation produced by hypothalamus with kidney injury [24]. Thus, serum CRE, BUN, TP, and PRO and urinary hyaline casts and low-transitional epithelium showed an immediate response with high sensitivity to kidney injury and hence could be used in the early diagnosis for GM-induced kidney injury. However, for some biomarkers that are not very specific, the simultaneous detection of several biomarkers could aid in accurate diagnosis. Moreover, our detection methods were simple, routine, convenient, and on cost-effective analyzers, which means it could be used in primary medical institutions for accurate diagnosis and effective monitoring of early kidney injury. According to Slater et al., investment in the field of diagnostic testing techniques can help save lives and help end the diseases in developing countries [25].

In recent years, new biomarkers for kidney injury such as kidney injury molecule-1 (KIM-1) and neutrophil gelatinase-associated lipocalin (NGAL) have been extensively studied [26–32]. KIM-1, also known as T-cells immunoglobulin mucin-1 (TIM-1), is a type-I transmembrane glycoprotein. In healthy kidneys, KIM-1 is undetectable; increased expression of this protein is found at very high levels on the apical membrane of proximal tubule cells after ischemic and nephrotoxic injury. KIM-1 expression is absent in the glomerulus, peritubular interstitial cells, and inner medullary cells [8, 33, 34]. NGAL is a protein belonging to the lipocalin superfamily initially found in activated neutrophils, in accordance with its role as an innate antibacterial factor. NGAL is secreted by the gastrointestinal tract, respiratory tract, and the kidney [35–37]. Research has shown that, after 2 h of ischemia, NGAL could be detected in urine samples, and its level was positively correlated with the duration of ischemia [36]. In addition, the abnormal expression of KIM-1 and NGAL may be related to other diseases as well, and hence, the mechanisms need to be further studied and confirmed. Furthermore, these molecules are detected by ELISA and RT-PCR, which are expensive and have a high demand on equipment and personnel, thereby making it unsuitable for routine use, especially in hospitals or areas without advanced facilities.

## 5. Conclusion

To summarize, serum CRE, BUN, and TP, urine PRO, and urinary hyaline casts and low-transitional epithelium showed an immediate and highly sensitive response to kidney injury, and the combined application of the above methods is helpful for definitive diagnosis in its early stage. Moreover, the detection methods were simple, routine, convenient, and on cost-effective analyzers, which means that it could be used in primary medical institutions for accurate diagnosis and effective monitoring of early kidney injury.

## Figures and Tables

**Table 1:** Serum levels of biomarkers in rats treated with different GM dosage and duration.

Items	Dosage (mg·kg^−1^)	Duration			
		Day 1	Day 3	Day 7	Day 14
TP (g·L^−1^)	0	83.430 ± 4.968^c^	86.218 ± 1.918^c^	87.352 ± 1.981^c^	87.933 ± 0.443^b^
	4	75.944 ± 2.772^b^	77.012 ± 2.534^b^	75.588 ± 2.320^c^	75.756 ± 0.054^a^
	20	73.333 ± 3.565^ab^	75.103 ± 2.558^b^	72.604 ± 1.892^b^	73.739 ± 2.828^a^
	100	70.749 ± 5.273^a^	64.221 ± 2.961^a^	63.169 ± 2.813^a^	—

ALB (g·dL^−1^)	0	33.975 ± 2.602^b^	39.374 ± 2.500^c^	40.109 ± 2.729^c^	41.122 ± 2.879^b^
	4	29.283 ± 2.405^a^	28.406 ± 1.810^b^	28.224 ± 1.937^b^	30.250 ± 1.743^a^
	20	28.360 ± 2.866^a^	26.942 ± 2.191^ab^	27.260 ± 1.852^b^	28.346 ± 0.810^a^
	100	27.412 ± 2.767^a^	24.628 ± 1.817^a^	22.130 ± 2.143^a^	—

CRE (*μ*mol·L^−1^)	0	179.056 ± 10.532^d^	178.355 ± 16.177^d^	170.954 ± 14.942^d^	188.069 ± 7.859^c^
	4	204.523 ± 9.235^c^	258.298 ± 10.460^c^	258.892 ± 12.464^c^	257.4305 ± 5.380^b^
	20	225.297 ± 15.217^b^	285.645 ± 15.434^b^	290.219 ± 11.497^b^	288.910 ± 12.692^a^
	100	250.534 ± 23.519^a^	331.240 ± 24.755^a^	358.339 ± 30.563^a^	—

BUN (mmol·L^−1^)	0	1.956 ± 0.335^c^	1.125 ± 0.355^c^	1.144 ± 0.252^d^	1.665 ± 0.301^c^
	4	3.023 ± 0.256^b^	4.085 ± 0.156^b^	5.286 ± 0.659^c^	6.168 ± 0.560^b^
	20	4.797 ± 0.675^ab^	6.399 ± 0.747^a^	7.713 ± 0.356^b^	8.176 ± 0.522^a^
	100	5.020 ± 0.380^a^	7.063 ± 0.503^a^	13.251 ± 0.399^a^	—

UA (*μ*mol·L^−1^)	0	268.261 ± 15.242^b^	267.636 ± 28.612^b^	265.544 ± 16.261^b^	275.924 ± 9.168^b^
	4	280.063 ± 30.455^ab^	283.889 ± 22.133^ab^	294.637 ± 13.512^b^	320.956 ± 20.945^a^
	20	295.234 ± 29.676^ab^	291.494 ± 22.681^ab^	301.259 ± 18.651^b^	329.063 ± 40.164^a^
	100	300.157 ± 34.472^a^	306.532 ± 37.724^a^	343.146 ± 37.630^a^	—

Values are expressed as mean ± SD for five rats in each group.

Groups: control (0 mg·kg^−1^) group, 4 mg·kg^−1^ GM group, 20 mg·kg^−1^ GM group, and 100 mg·kg^−1^ GM group.

In each column, same letters indicate no significant difference (*P* > 0.05), with significant difference (*P* < 0.05).

**Table 2:** Urinary pH and SG of rats treated with different GM dosage and duration.

Items	Dosage (mg·kg^−1^)	Duration			
		Day 1	Day 3	Day 7	Day 14
pH	0	7.625 ± 0.899	7.833 ± 0.707	7.500 ± 1.041^b^	7.750 ± 0.354
	4	7.417 ± 0.970	7.500 ± 0.764	6.833 ± 0.707^ab^	6.786 ± 0.735
	20	7.500 ± 0.524	7.000 ± 0.758	6.643 ± 0.703^ab^	6.570 ± 0.418
	100	7.250 ± 0.641	6.800 ± 0.780	5.000 ± 1.140^a^	—

SG	0	1.022 ± 0.004	1.025 ± 0.003	1.023 ± 0.003	1.024 ± 0.004
	4	1.023 ± 0.005	1.027 ± 0.007	1.028 ± 0.004	1.027 ± 0.004
	20	1.025 ± 0.005	1.028 ± 0.002	1.028 ± 0.003	1.028 ± 0.003
	100	1.027 ± 0.004	1.030 ± 0.005	1.030 ± 0.005	—

Values are expressed as mean ± SD for five rats in each group.

Groups: control (0 mg·kg^−1^) group, 4 mg·kg^−1^ GM group, 20 mg·kg^−1^ GM group, and 100 mg·kg^−1^ GM group.

^a^ *P* < 0.05 versus 0 mg·kg^−1^ groups.

^b^ *P* < 0.05 versus 100 mg·kg^−1^ GM groups.

**Table 3:** Change in urinary PRO in rats treated with different GM dosage and duration.

Dosage (mg·kg^−1^)	Duration							
	Day 1		Day 3		Day 7		Day 14	
0	−	33.33%	−	57.14%	−	71.43%	−	67.14%
	+−	66.67%	+−	42.86%	+−	28.57%	+−	32.86%
	+	0	+	0	+	0	+	0
	2+	0	2+	0	2+	0	2+	0
	3+	0	3+	0	3+	0	3+	0

4	−	20.00%	−	14.29%	−	0	−	0
	+−	40.00%	+−	14.29%	+−	25.00%	+−	40.00%
	+	20.00%	+	57.13%	+	62.50%	+	40.00%
	2+	20.00%	2+	14.29%	2+	12.50%	2+	20.00%
	3+	0	3+	0	3+	0	3+	0

20	−	14.29%	−	12.50%	−	0	−	0
	+−	42.85%	+−	37.50%	+−	28.57%	+−	20.00%
	+	28.57%	+	25.25%	+	42.86%	+	20.00%
	2+	14.29%	2+	25.25%	2+	28.57%	2+	40.00%
	3+	0	3+	0	3+	0	3+	20.00%

100	−	12.50%	−	0	−	0	−	
	+−	25.00%	+−	14.29%	+−	0		
	+	37.50%	+	42.85%	+	16.67%		
	2+	25.00%	2+	28.57%	2+	33.33%		
	3+	0	3+	14.29%	3+	50.00%		

Groups: control (0 mg·kg^−1^) group, 4 mg·kg^−1^GM group, 20 mg·kg^−1^ GM group, and 100 mg·kg^−1^ GM group.

**Table 4:** Kidney coefficient of rats treated with different GM dosage and duration.

Kidney coefficient	Dosage (mg·kg^−1^)	Duration			
		1st day	3rd day	7th day	14th day
Left kidney	0	0.449 ± 0.088	0.351 ± 0.006	0.385 ± 0.059	0.360 ± 0.005
	4	0.385 ± 0.018	0.397 ± 0.029	0.421 ± 0.037	0.417 ± 0.069
	20	0.411 ± 0.074	0.378 ± 0.060	0.438 ± 0.038	0.382 ± 0.050
	100	0.424 ± 0.007	0.377 ± 0.047	0.440 ± 0.076	0.486 ± 0.038

Right kidney	0	0.428 ± 0.037	0.340 ± 0.029	0.389 ± 0.034	0.387 ± 0.032
	4	0.357 ± 0.010	0.395 ± 0.054	0.464 ± 0.63	0.432 ± 0.073
	20	0.380 ± 0.025	0.373 ± 0.022	0.476 ± 0.025	0.383 ± 0.043
	100	0.389 ± 0.035	0.389 ± 0.056	0.454 ± 0.101	0.531 ± 0.074

Values are expressed as mean ± SD for five rats in each group.

Groups: control (0 mg·kg^−1^) group, 4 mg·kg^−1^ GM group, 20 mg·kg^−1^ GM group, and 100 mg·kg^−1^ GM group.
